# Genomic Sequencing and Functional Analysis of the Ex-Type Strain of *Malbranchea zuffiana*

**DOI:** 10.3390/jof10090600

**Published:** 2024-08-24

**Authors:** Alan Omar Granados-Casas, Ana Fernández-Bravo, Alberto Miguel Stchigel, José Francisco Cano-Lira

**Affiliations:** Mycology Unit, School of Medicine, Universitat Rovira i Virgili, C/ Sant Llorenç 21, 43201 Reus, Spain; alanomar.granados@urv.cat (A.O.G.-C.); jose.cano@urv.cat (J.F.C.-L.)

**Keywords:** genes, genome assembly, keratin degradation, *Malbranchea zuffiana*, *Onygenales*, phylogenomics, proteins

## Abstract

*Malbranchea* is a genus within the order *Onygenales* (phylum Ascomycota) that includes predominantly saprobic cosmopolitan species. Despite its ability to produce diverse secondary metabolites, no genomic data for *Malbranchea* spp. are currently available in databases. Therefore, in this study, we obtained, assembled, and annotated the genomic sequence of the ex-type strain of *Malbranchea zuffiana* (CBS 219.58). For the genomic sequencing, we employed both the Illumina and PacBio platforms, followed by hybrid assembly using MaSuRCA. Quality assessment of the assembly was performed using QUAST and BUSCO tools. Annotation was conducted using BRAKER2, and functional annotation was completed with InterProScan. The resulting genome was of high quality, with a size of 26.46 Mbp distributed across 38 contigs and a BUSCO completion rate of 95.7%, indicating excellent contiguity and assembly completeness. A total of 8248 protein-encoding genes were predicted, with functional annotations assigned to 73.9% of them. Moreover, 82 genes displayed homology with entries in the Pathogen Host Interactions (PHI) database, while 494 genes exhibited similarity to entries in the Carbohydrate-Active Enzymes (CAZymes) database. Furthermore, 30 biosynthetic gene clusters (BGCs) were identified, suggesting significant potential for the biosynthesis of diverse secondary metabolites. Comparative functional analysis with closely related species unveiled a considerable abundance of domains linked to enzymes involved in keratin degradation, alongside a restricted number of domains associated with enzymes engaged in plant cell wall degradation in all studied species of the *Onygenales*. This genome-based elucidation not only enhances our comprehension of the biological characteristics of *M. zuffiana* but also furnishes valuable insights for subsequent investigations concerning *Malbranchea* species and the order *Onygenales*.

## 1. Introduction

The genus *Malbranchea* was established by Saccardo in 1882, in honor of the botanist and mycologist Alexandre François Malbranche [1]. The type species, *Malbranchea pulchella*, is characterized by the production of hyaline, branched, and curved hyphae, as well as yellowish, oblong-cylindrical conidia, truncated on both ends [1]. In 1964, Cooney and Emerson distinguished two *Malbranchea* species based on their optimal growth temperature, with *M. pulchella* classified as mesophilic and *Malbranchea sulfurea* as thermophilic [2]. Subsequently, a total of twelve species of *Malbranchea* were identified in 1976, and a connection was made with the genus *Auxarthron* as their sexual counterpart. This association was established on *Auxarthron conjugatum* forming a malbranchea-like asexual morph, while *Malbranchea albolutea* produces an auxarthron-like sexual stage [3].

In 2002, Sigler et al. elucidated the association of *Malbranchea filamentosa* and *Malbranchea albolutea* with the genus *Auxarthron* through in vitro matting experiments and molecular analysis [4]. Subsequently, Rodríguez-Andrade et al. (2021) conducted a comprehensive phylogenetic analysis of malbranchea-like clinical fungal strains, proposing the synonymy between *Auxarthron* and *Malbranchea* based on their findings [5]. In the latest phylogenetic revision of the order *Onygenales* by Kandemir et al. (2023), among other taxonomic novelties, the erection of the family *Malbrancheaceae* was proposed [6]. More recently, Torres-Garcia et al. increased the list of known species of *Malbranchea* by finding three more from river sediments in Spain [7]. According to the Index Fungorum (https://www.indexfungorum.org/names/Names.asp, accessed on 20 October 2023), *Malbranchea* comprises 44 records; however, several of these names have become obsolete, such as *Malbranchea bolognesii-chiurco* and *Malbranchea sulfurea*. Furthermore, certain species have been reclassified into other genera; for example, *Malbranchea gypsea* is now classified under *Spiromastigoides*, *Malbranchea pulveracea* under *Botryobasidium,* and *Malbranchea sclerotica* under *Arachnomyces* [5,8].

*Malbranchea* species exhibit a ubiquitous distribution worldwide, isolated from diverse environmental reservoirs, including soil, air, dung, decaying plant material, and various animal hosts [9,10,11,12,13]. Prior investigations have identified distinct species within this genus from clinical specimens from both human and animal subjects [14,15,16]. Notably, certain species within the genus exhibit thermotolerance or thermophily, features that facilitate the colonization of host tissues [2,3,5]. Nevertheless, their potential as pathogenic agents, akin to other taxa within the order *Onygenales*, remains a subject of ongoing debate.

Routine identification of *Malbranchea* spp. from clinical samples, as with other members of the *Onygenales*, relies on a single strain and the phenotypic characterization of their reproductive structures. The past two decades have seen rapid advancements in molecular-based identification techniques, notably MALDI-TOF, which have experienced increasing popularity and adoption [16,17,18,19]. Despite these advancements, other powerful techniques for fungal identification based on gene sequencing remain relatively underutilized. Nonetheless, Hubka et al. (2013) identified *Malbranchea ostraviensis* (formerly *Auxarthron ostraviense*) and *Malbranchea umbrina* (syn. *Auxarthron umbrinum*) as etiologic agents of onychomycosis [20]. Later, *Malbranchea albolutea* (syn. *Auxarthron alboluteum*) was involved in a non-dermatophytic case of onychomycosis [21]. In 2020, Tang et al. reported the finding of *Malbranchea flocciformis* through metagenomics (NGS) analysis in a skin sample of a dog clinically affected by a fungal infection [22]. More recently, Rodríguez-Andrade et al. (2021) identified, by sequencing of the internal transcribed spacer (ITS) and of the D1 and D2 domains (D1−D2) of the large subunit (LSU) of the rRNA, twenty-two malbranchea-like strains from human and animal sources [5]. Of these, 15 belonged to different species of the genus *Malbranchea*: *Malbranchea umbrina*, four strains; *Malbranchea aurantiaca*, two strains; *Malbranchea albolutea*, two strains; *Malbranchea conjugata*, two strains; and one strain each of *Malbranchea flocciformis*, *Malbranchea gymnoascoides*, *Malbranchea multiseptata*, *Malbranchea stricta*, and *Malbranchea zuffiana* [5].

Like other fungi, *Malbranchea* spp. produce a wide variety of molecules of potential biotechnological interest [22,23,24]. One notable molecule, isolated and purified from a strain of *Malbranchea pulchella* var. *sulphurea*, is an enzyme with serine protease activity, which is also thermostable [23]. This research opened the door to many studies conducted to identify various enzymes and secondary metabolites (SMs) produced by *Malbranchea* species (Table 1).

Currently, the National Center for Biotechnology Information (NCBI) nucleotide database contains only a single genome of a *Malbranchea* species, specifically, *Malbranchea cinnamomea* strain FCH_10_5. However, the taxonomic circumscription of this species is difficult because of the lack of a living strain derived from the type material. Furthermore, phylogenetically informative nucleotide sequences from these strains align with different fungal families and orders when analyzed using BLAST (https://blast.ncbi.nlm.nih.gov, accessed 20 October 2023) [AN].

Consequently, due to the absence of reliable *Malbranchea* spp. genomes in public genomic databases, the main objective of our work was to sequence, assemble, and annotate the complete genome of the ex-type strain of *M. zuffiana*, CBS 219.58, and conduct a comparative functional analysis with phylogenetically closely related taxa.

## 2. Material and Methods

### 2.1. DNA Extraction for Genome Sequencing

The genomic DNA of *Malbranchea zuffiana* CBS 219.58 was extracted using a modified protocol of the DNeasy^®^ Plant Mini Kit (Qiagen, Hilden, Germany). Subsequently, the DNA underwent quality control using Nanodrop 2000 (Thermo Scientific, Madrid, Spain) and the Qubit 2.0 Fluorometer (Invitrogen, Carlsbad, CA, USA). The extracted DNA was then sequenced using both short and long reads, employing the Illumina NovaSeq6000 sequencing system (Illumina, San Diego, CA, USA) with 150 PE (150 × 2 bp), as well as the PacBio Sequel I system (Pacific Biosciences of California, Inc., Menlo Park, CA, USA), performed by Macrogen (Seoul, Republic of Korea).

For the Illumina sequencing, the genomic DNA library was prepared using the Truseq Nano DNA library. For PacBio sequencing, a 10 kb insert library was prepared using SMARTbell Express. To ensure the quality of Illumina reads, the FastQC v0.11.9 tool was employed [43] to visually inspect the quality of the reads and identify low-quality ones. Subsequently, Trimmomatic v0.39 [44] was used to trim adapters and low-quality reads, resulting in a dataset of high-quality sequencing data. The high-quality reads were then subjected to a hybrid assembly using MaSuRCA v4.0.5 [45] software with default settings. The resulting draft assembly was subsequently refined by POLCA (a tool from MaSuRCA v.4.0.5) using the Illumina short-read data. The quality of the assembly was evaluated using the Quality Assessment Tool (QUAST) v5.1.0rc.1 [46] and the Benchmarking Universal Single-Copy Orthologs (BUSCO) v5.3.1 [47], using the *Onygenales*_odb10 lineage dataset. Additionally, the ribosomal RNAs were predicted using Barrnap v0.9 [48], while the transfer RNA sequences were predicted with tRNAscan-SE v2.0.9 [49].

### 2.2. Genome Assembly and Annotation

The BRAKER2 v2.1.6 pipeline was used for gene prediction, utilizing the GeneMark-ET and AUGUSTUS packages [50]. Functional annotation was performed using InterProScan v5.55-88.0 [51], with the PFAM and SUPERFAMILY options, considering only assignments with an E-value equal to or higher than 1.00 × 10^−5^. Later, the functional classification in Clusters of Orthologous Groups (COGs) was executed using the COGclassifier v1.0.5 tool (https://github.com/moshi4/COGclassifier, accessed 9 July 2023). Subsequently, the identification of genes related to carbohydrate-active enzymes (CAZymes) was conducted through the annotation tool Run_dbCAN v3 [52], with the DIAMOND option and default settings. Pathogenicity-related genes were detected using DIAMOND v2.0.15 [53] utilizing the pathogen–host interaction (PHI) database [54] through a Blastp search, with the following parameters: E-value of 1.00 × 10^−5^, max-target seqs of 1, 80% identity, amino acid length ≥ 100, 60% query coverage, and 60% subject coverage. The biosynthetic gene clusters (BGCs) prediction was performed using the antiSMASH v6.1.1 tool [55] with default settings. Finally, to identify homologous genes between *M. zuffiana* CBS 219.58 and those involved in malbrancheamide production, the gene sequences of *malA*, *malB*, *malC*, *malD*, *malE*, *malF*, and *malG* from *Malbranchea aurantiaca* RRC1813 were obtained from the UniProt database (accessed 3 May 2023). Subsequently, a search to identify these genes was conducted using a local Blastp [56] with the default settings.

### 2.3. Phylogenetic and Functional Comparative Analysis

To determine the phylogenetic placement of the strain CBS 219.58 and to conduct a comparative functional annotation, the protein sequences of the phylogenetically closest species were downloaded from the NCBI database (accessed 4 May 2023) for *Uncinocarpus reesii* UAMH 1704 (GenBank Assembly Accession (GCA) GCA_000003515.2), *Coccidioides immitis* RS (GCA_000149335.2), *Coccidioides posadasii* C735 (GCA_000151335.1), *Chrysosporium keratinophilum* CBS 104.62 (GCA_029850275.1), *Aphanoascus verrucosus* IHEM 4434 (GCA_014839905.1), *Nannizzia gypsea* CBS 118893 (GCA_000150975.2), *Trichophyton rubrum* CBS 118892 (GCA_000151425.1), *Trichophyton benhamiae* CBS 112371 (GCA_000151125.2), *Microsporum canis* CBS 113480 (GCA_000151145.1), *Paracoccidioides brasiliensis* PB01 (GCA_000150735.2), *Histoplasma capsulatum* G186AR (GCA_000150115.1), *Arachnomyces peruvianus* gpAraPeru1.1 (GCA_949709995.1), *Aspergillus fumigatus* Af293 (GCA_000002655.1), *Aspergillus nidulans* FGSC A4 (GCA_000011425.1), and *Aspergillus flavus* NRRL3357 (GCA_014117465.1).

Initially, to perform the phylogenetic analysis the orthologs among the analyzed strains were predicted using Orthofinder v2.5.5 [57], utilizing default settings. From these, we randomly selected 50 single-copy orthogroups (all with more than 200 amino acids); subsequently, the sequences were aligned using MAFFT v7.5 [58]. Poorly aligned positions were then trimmed using Gblocks v0.91b. The resulting alignments were finally concatenated into a super-alignment. The best protein substitution model was determined using ModelTest-NG x.y.z [59], followed by a maximum likelihood phylogenetic analysis using RAxML-NG v0.9.0 [60] with 1000 bootstrap replicates. For the functional comparative analysis, all the downloaded proteins were annotated using InterProScan with the PFAM option, as previously described.

## 3. Results and Discussion

### 3.1. Genome Assembly and Evaluation

A total of 1.61 Gb and 7.43 Gb of raw data were generated using the Illumina and PacBio Sequel I platforms, respectively. The hybrid assembly of the genome of the strain CBS 219.58 comprised 26.46 Mb, with a GC content of 49.06% and 38 contigs. Additionally, the genome showed an N50 of over 1.5 Mb, an L50 of 7, and 0.00 Ns per 100 kbp. The BUSCO assessment of genome completeness predicted 95.7% (4653 of 4862) of complete BUSCOs from the *Onygenales* dataset, indicating excellent contiguity and assembly completeness. Other general features of the genome are listed in Table 2.

The genome of *M. zuffiana* CBS 219.58 was slightly larger than most of the genomes of closely related species of the *Onygenaceae* family, except for the genomes of *Coccidioides posadasii* C735 (27.01 Mb), *Coccidioides immitis* RS (29.01 Mb), and *Nannizziopsis barbatae* USC001 (31.54 Mb), the species considered as human and animal pathogen [61,62]. It is also noteworthy that this genome has a lower number of contigs compared to other genomes of the same order, which is directly attributed to the utilization of long-read sequencing technology (PacBio). This technology has significantly contributed to reducing the number of contigs and gaps in the genome. Furthermore, 42 and 177 rRNAs and tRNAs, respectively, were successfully identified. Regarding the tRNAs, 158 decoded the 20 standard amino acids, one tRNA was classified as an undetermined isotype, and 18 were predicted as pseudogenes.

### 3.2. Genome Annotation

**Figure 1:**
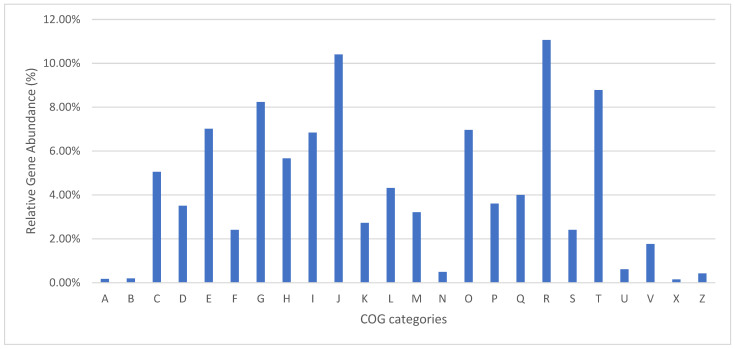
Functional annotation of CBS 219.52. A—RNA processing and modification; B—chromatin structure and dynamics; C—energy production and conversion; D—cell cycle control, cell division, chromosome partitioning; E—amino acid transport and metabolism; F—nucleotide transport and metabolism; G—carbohydrate transport and metabolism; H—coenzyme transport and metabolism; I—lipid transport and metabolism; J—translation, ribosomal structure, and biogenesis; K—transcription; L—replication, recombination, and repair; M—cell wall/membrane/envelope biogenesis; N—cell motility; O—post-translational modification, protein turnover, chaperones; P—inorganic ion transport and metabolism; Q—secondary metabolite biosynthesis, transport, and catabolism; R—general function prediction only; S—function unknown; T—signal transduction mechanisms; U—intracellular trafficking, secretion, and vesicular transport; V—defense mechanisms; X—mobilome: prophages, transposons; Z—cytoskeleton.

The ab initio annotation approach for the genome predicted a total of 37,353 coding sequences (CDSs) and 8248 proteins. According to the functional COG classification, 4077 genes (49.43% of the total) were categorized into 24 different COG categories. The top three COG categories, in order, were “General function prediction only” (451 proteins), “Translation, ribosomal structure and biogenesis” (424 proteins), and “Signal transduction mechanisms” (358 proteins). However, 98 proteins were classified as “Function unknown” (Figure 1). Subsequent functional annotation with InterProScan led to the identification of 3579 PFAM groups with 6099 (73.9%) annotated proteins, and 835 SUPERFAMILY groups with 4815 (58.37%) annotated proteins. However, 2149 and 3433 proteins found no significant annotation with the PFAM and SUPERFAMILY options, respectively. In the case of the PFAM option, the groups with a higher number of identifications were “Ankyrin repeats (3 copies)” (224 proteins), “WD domain, G-beta repeat” (160 proteins), “Protein kinase domain” (156 proteins), “Major Facilitator Superfamily” (128 proteins), and “Fungal specific transcription factor domain” (113 proteins). Most of these proteins were previously associated with signal transduction, transcription regulation, cell cycle regulators, and apoptosis, as well as transporters for a large group of substrates, including ions, carbohydrates, lipids, amino acids, and peptides, among others [63,64,65].

**Figure 2:**
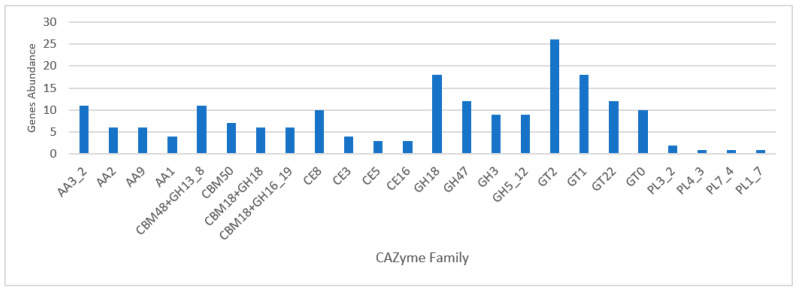
Most prevalent CAZyme families (in number of genes) identified per class in the strain CBS 219.58.

CAZymes represent a group of enzymes responsible for the biosynthesis and degradation of carbohydrates and are categorized into different families according to their sequence similarities and catalytic activities [66]. Several studies have shown the remarkable diversity of CAZymes produced in fungi, including ascomycetes and basidiomycetes. These enzymes play a fundamental role in fungal physiology, in processes such as cell wall remodeling, nutrient acquisition, and interactions with the environment [67,68,69]. A total of 497 genes from *M. zuffiana* CBS 219.58 were associated with CAZymes. Of these, 198 genes encode glycoside hydrolases (GHs), distributed in 73 different families; 165 genes encode glycosyl transferases (GTs), belonging to 37 families; 60 genes encode carbohydrate-binding modules (CBMs), in 24 families; 45 genes encode auxiliary activities (AAs), distributed in 15 families; 24 genes encode carbohydrate esterases (CEs) in eight families; and finally, five genes encode polysaccharide lyases (PLs), distributed in four families. The most abundant CAZyme families per group are presented in Figure 2. According to our findings, the strain CBS 219.58 possesses a CAZyme profile comparable to that of saprophytic fungi, with a total number of over 200 CAZymes, and lacking CAZymes from the CE11, GH73, GH80, and GH82 groups [70]. Additionally, we identified some groups of CAZymes related to cellulolytic activity, such as GH5 and GH3, which were also detected in the PFAM analysis [71].

The potential pathogenesis-related genes were subsequently identified. A total of 82 genes showed homology with those of the Pathogen–Host Interaction (PHI) database. These homologous genes spanned across 17 different species, with *Aspergillus fumigatus* having the highest number of homologous genes (27 genes), followed by *Fusarium graminearum* with 17 genes, and *Magnaporthe oryzae* with 15 genes. Categorizing these homologous genes revealed three predominant groups: “Reduced virulence” with 36 genes (43% of the total), “Unaffected pathogenicity” with 16 genes (19.5%), and both “Mixed” and “Lethal” with 12 genes each (14.6%). Based on the Clusters of Orthologous Groups (COGs) classification, a substantial portion of the PHI-associated genes were linked to “General function prediction only”, “Signal transduction mechanisms”, and “Amino acid transport and metabolism”. This classification highlights the multifaceted nature of the identified genes, indicating their involvement in a diverse array of cellular functions crucial for pathogenic interaction. Furthermore, our investigation revealed the presence of the *MoMca2* (MGG_13530) gene, which encodes Metacaspase-1 in *Magnaporthe oryzae*. This gene plays a crucial role in regulating stress responses and the pathogenicity of this fungus [72]. Another significant finding was the identification of the *FgHSP90* gene of *Fusarium graminearum*, responsible for encoding the ubiquitous chaperone heat shock protein 90. This gene is essential for the development and virulence of the fungus and has been previously identified in other pathogenic fungi, such as *Candida albicans* and *Aspergillus fumigatus* [73,74,75].

### 3.3. AntiSMASH

The genes involved in synthesizing and regulating the production of secondary metabolites are typically clustered together, in what are commonly referred to as biosynthetic gene clusters (BGCs) [76,77]. The analysis conducted using AntiSMASH identified 30 BGCs, which were categorized into seven distinct types, including non-ribosomal peptide synthetase (NRPS) (*n* = 7), Type I PKS (polyketide synthase) (T1PKS) (*n* = 5), NRPS-like fragment (NRPS-like) (*n* = 4), terpene (*n* = 4), indole (*n* = 2), fungal post-translationally modified peptide product-like (fungal-RiPP-like) (*n* = 2), and cluster hybrids (*n* = 6). The three predominant groups were NRPS, boasting 7 BGCs, followed by hybrid clusters with 6 BGCs, and T1PKS with five clusters. Regarding previously reported BGCs, the genus *Aspergillus* exhibited the highest number of similarities, with three clusters. This was followed by the genera *Penicillium* and *Fusarium*, each showing similarities with two clusters. Remarkably, four BGCs exhibited a 100% similarity to previously documented clusters, specifically, enniatin, epichloenin A, clavaric acid, and choline (Supplementary Table S1 and Figure S1).

The enniatins are mycotoxins of significant dietary importance, primarily associated with the genus *Fusarium*, although they have also been identified in fungi such as *Verticillium* and *Halosarpheia*. At present, more than 29 enniatin analogs have been described, and identified in various foods, most commonly in processed and unprocessed grains [78,79]. As for epichloenin A, this compound functions as an extracellular siderophore synthesized by the systemic endophytic fungus *Epichloë festucae*, playing a crucial role in the mutualistic interaction between the fungus and the plant (*Epichloë festucae*/*Lolium perenne*) [80]. Clavaric acid is considered a triterpenoid compound with antitumor properties, produced by the basidiomycete fungus *Hypholoma sublateritium*. This compound has been found to inhibit human ras-farnesyl transferase [81]. It has recently been identified in the closely related species *Chrysosporium keratinophilum* [82]. Finally, regarding choline, its direct role as a secondary metabolite is not well understood [83]; however, its importance as an essential metabolite for the development and growth of fungi is widely recognized [84]. Additionally, among the 30 BGCs found, nine showed similarities (ranging from 9% to 40%) with previously reported clusters. The matches include equisetin (18% similarity), trypacidin (35%), griseofulvin (9%), cheatoglobosin P (25%), trichobrasilenol (40%), squalestatin S1 (40%), HEx-pks23 polyketide (33%), solanapyrone (40%), and communesin A (25%). Remarkably, 17 BGCs exhibited no resemblance to any previously described BCG, suggesting the potential production of novel metabolites. While these findings provide valuable insights into BCG diversity and possible implications, further research at both functional and molecular levels is imperative to elucidate the specific roles of these identified BGCs.

**Figure 3:**
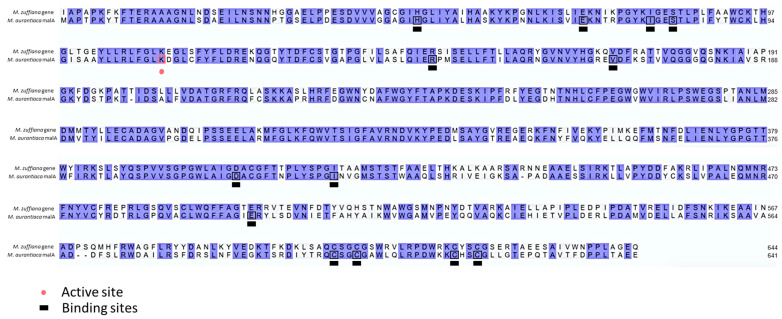
Sequence alignment of amino acid sequences of malA protein from *Malbranchea aurantiaca* RRC1813 and *Malbranchea zuffiana* CBS 219.58.

The synthesis of malbrancheamide, a dichlorinated fungal alkaloid that acts as a calmodulin inhibitor, was previously described in *Malbranchea aurantiaca* RRC1813 and *Malbranchea graminicola* 086937A [24,85]. Like other SMs, malbrancheamide is encoded by a BGC, consisting of a total of seven genes: *malA*, coding for a flavin-dependent halogenase; *malB*, prenyltransferase; *malC*, for short-chain dehydrogenase/reductase; *malD*, which encodes an NmrA-like family domain-containing oxidoreductase; *malE*, for prenyltransferase; and *malF*, for an FAD-linked oxidoreductase [86]. However, despite this cluster being registered in the antiSMASH database, no results associated with this BGC were observed in the genome of *M. zuffiana* CBS 219.58. Subsequently, through the Blastp analysis, a potential homology was identified between a protein from *M. zuffiana* and the protein resulting from *malA*. This homology exhibited an E-value of 0.0 and a 68% identity, also having identical active sites and binding sites, reinforcing the homology among them (Figure 3). Nevertheless, no homologous genes related to the remaining genes associated with malbrancheamide synthesis were identified. Due to the unavailability in the databases of nucleotide sequences of phylogenetically informative genes or genomic regions (ITS-LSU), it was not possible to perform a direct comparison of *M. aurantiaca* RRC1813 and *M. graminicola* 086937A with the strain of *M. zuffiana* sequenced by us. Such a comparison could have yielded crucial insights into the phylogenetic relationships and genetic composition between these strains. Future investigations could be focused on obtaining and analyzing a greater number of reference genomes of this genus, thereby enriching our understanding of the genus *Malbranchea* and enabling a more comprehensive evaluation of its genetic and phylogenetic diversity.

### 3.4. Phylogenomics

The Orthofinder analysis resulted in a total of 143,830 identified proteins, with 132,766 proteins (92.3% of the total) assigned to 11,100 orthologous groups. It is noteworthy that only 3760 orthogroups were present in all the species, and of these, 2357 consisted entirely of single-copy orthologs. The final alignment exhibited a total length of 26,071 amino acids (including gaps), with 42.65% representing invariant sites, 57.35% variable sites, and 44.08% being parsimony informative sites.

**Figure 4:**
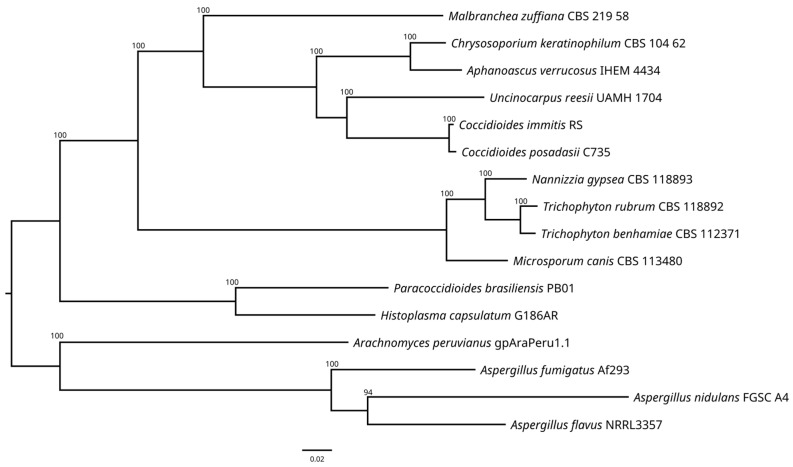
Maximum likelihood (ML) phylogenetic tree of fifty concatenated single-copy orthologous using RAxML with the JTT+I+G4+F model and 1000 ultrafast bootstrap replicates.

The resulting phylogenetic tree (Figure 4) revealed the formation of five highly supported clades. The first of them contains only the genome of *M. zuffiana*, which represents the only strain belonging to the family *Malbrancheaceae*. The second encompasses the strains of the family *Onygenaceae*, including species of *Chrysosporium* (as the asexual counterpart of the genus *Aphanoascus*), *Coccidioides,* and *Uncinocarpus*. The third consists of strains belonging to the family *Arthrodermataceae*, including species of the genera *Microsporum*, *Nannizzia*, and *Trichophyton*. The fourth encompasses the family *Ajellomycetaceae*, and includes the genera *Histoplasma* and *Paracoccidioides*. Finally, the fifth clade comprises strains of the genera *Aspergillus* (order Eurotiales) and *Arachnomyces* (order *Arachnomycetales*), used as the outgroup. The phylogenetic analysis revealed a clear separation of *M. zuffiana* CBS 219.58 from the rest of the members of the families mentioned above. These results are consistent with those previously obtained by Kandemir et al. [6], who proposed the erection of the family *Malbrancheaceae* among other taxonomic novelties.

The results of the PFAM analysis showed that strains belonging to the families *Arthodermataceae* (*Microsporum canis*, *Nannizzia gypsea*, *Trichophyton benhamiae*, *Trichophyton rubrum*), *Malbrancheaceae* (*M. zuffiana*), and *Onygenaceae* (*Aphanoascus verrucosus, Chrysosporium keratinophilum, Coccidioides immitis, Coccidioides posadasii, Uncinocarpus reesii*) have a significantly high number of domains associated with endoproteases and exoproteases related to keratin degradation. In contrast, strains of the family *Ajellomycetaceae*, which includes *Histoplasma capsulatum* and *Paracoccidioides brasiliensis*, have significantly lower levels of these domains. Concerning the domains associated with oligopeptidases, a notable uniformity was observed across the genomes within the *Onygenales*, as is detailed in Table 3. It is noteworthy that CBS 219.58, belonging to the *Malbrancheaceae*, demonstrated a similar profile of domains related to keratin degradation as was observed in members of the *Onygenaceae*. This can be attributed to the close phylogenetic relationship between both families [5,6].

The abundance of domains associated with keratin degradation in *Arthrodermataceae*, *Malbrancheaceae,* and *Onygenaceae* can be directly linked to the environments in which these fungi normally develop, characterized by the constant presence of keratin substrates [5,87]. Moreover, in past times keratin degradation was a predominant physiological trait in this group of fungi [88,89]. In contrast, fungi belonging to the *Ajellomycetaceae* are typically animal pathogens, also producing systemic infections in humans. Nevertheless, their ecological niches remain either unknown or poorly understood. For *Histoplasma capsulatum*, its recognized natural reservoir is soils enriched in guano from birds or bats [90], while the natural reservoir remains unknown for *Paracoccidioides brasiliensis*, since its isolation from soil samples is exceptional [91]. Consequently, it can be assumed that the potential niches associated with the *Ajelllomycetaceae* are low in keratin content, suggesting that the selective pressure of the environment could be a factor contributing to the reduction in enzymes for keratin degradation.

Cellulose degradation was historically considered a distinctive characteristic of some species within the *Onygenales*, particularly the genera of the family *Myxotrichaceae*. However, a phylogenetic analysis conducted by Wang et al. in 2006 relocated this family within the class *Leotiomycetes*, leaving the taxa with cellulolytic activity out of the *Onygenales* [92]. Our results show that all the analyzed genomes of the *Onygenales* have a reduced number or lack of domains associated with plant cell wall-degrading enzymes, such as cellulose, pectin, and xylan, emphasizing their strong preference for substrates rich in keratin over plant material (Table 4).

Secondary metabolites are organic molecules whose synthesis is not essential for the normal development of the fungus, but their production is linked to environmental competition or adaptive response. SMs are mainly classified into four chemical families: nonribosomal peptides (NRPs), polyketides (PKs), NRP/PKS hybrids, and terpenoids [93,94]. While some of these SMs are harmful to humans, such as mycotoxins, many others have numerous beneficial applications, including antibiotics, antitumor agents, vitamins, and pigments, among others [95,96]. The PFAM analysis results of domains associated with secondary metabolite production revealed significant variability in the number of identified genes among the analyzed strains. Interestingly, there were similarities in values among closely related strains. So, *Microsporum canis* showed the highest number of genes (286) associated with SM production, closely followed by *Nannizzia gypsea* and *Trichophyton benhamiae*, both with 234 genes. Contrarily, *Paracoccidioides brasiliensis*, with 84 genes, and *Histoplasma capsulatum*, with 107 genes, displayed the lowest number of genes involved in the production of PKs, NRPs, and NRP/PK hybrids (Table 5).

Regarding the domains associated with terpene production, most strains exhibited a low number or absence of the “Terpene synthase family 2 C-terminal metal binding” domain. The highest value was observed in *M. zuffiana*, with six genes, followed by *Microsporum canis*, with five genes. Regarding the remaining terpene domains, specifically “Terpene synthase, N-terminal domain” and “Terpene synthase family, the metal binding domain”, the absence of these domains was observed in all the strains analyzed. Previous research has shown the crucial role of volatile terpenes produced by fungi in the interactions between endophytic fungi and host plants [97,98]. Since the fungal genomes we analyzed displayed a scarce number of genes related to terpene production, we assume that these fungi do not play an important role as plant pathogens or symbionts. Concerning CBS 219.58, this strain exhibited a profile of domains linked to the production of secondary metabolites that surpassed all other *Onygenaceae* members, being only lower than the members of the *Arthrodermataceae*. These findings highlight the significant potential of the *M. zuffiana* CBS 219.58 genome, which we sequenced, to produce secondary metabolites. Thus, further research is necessary for a more comprehensive understanding of its biosynthetic pathways and their consequential impact on its functional ecology.

## 4. Conclusions

In this study, we unveil the first sequenced and published genome of one ex-type strain of the genus *Malbranchea*, *Malbranchea zuffiana* CBS 219.58, employing a hybrid assembly strategy. The genome annotation and comprehensive genomic analysis yield fresh insights that enhance our comprehension of this genus and the order *Onygenales*. Moreover, elucidating biosynthetic gene pathways confirms its genetic capacity to produce secondary metabolites, suggesting significant biosynthetic potential for compounds of biotechnological interest. However, further analyses are necessary to validate the ab initio detection of promoters, coding or noncoding regions, and intron–exon junctions in the sequenced genome of *M. zuffiana* CBS 219.58.

## Figures and Tables

**Table 1:** Previously reported metabolites in *Malbranchea* species.

Species	Reported Metabolite [Reference]
*Malbranchea aurantiaca*	Malbrancheamide [24], Malbrancheamide B [25], Phytotoxins (1-hydroxy-2-oxoeremophil-1(10), 7(11),8(9)-trien-12(8)-olide and penicillic acid) [26], Premalbrancheamide [27]
*Malbranchea dendritica*	α-Glucosidase and Protein tyrosine phosphatase 1B (PTP-1B) [28]
*Malbranchea filamentosa*	Cytotoxic anthrasteroid glycosides, malsterosides A–C [29]
*Malbranchea flavorosea*	Polyketides (8-chloroxylarinol A, flavoroseoside) [30]
*Malbranchea graminicola*	Spiromalbramide and isomalbrancheamide B [31]
*Malbranchea pulchella*	β-glucosidase [32]
*Malbranchea pulchella* var. *sulfurea*	Antibiotic (Tf-26Vx) [33], β-xylosidase [34,35], Lipoamide dehydrogenase [36], Protease [37,38], Serine protease [39], Trehalase [40], Xylanase [41,42]

**Table 2:** Summary statistics of *de novo* assembly of the ex-type strain of *Malbranchea zuffiana*, CBS 219.58.

Parameter	Amount **
Illumina reads	8,179,242
PacBio subreads	609,002
# contigs (≥0 bp)	38
Largest contig	2,639,719
Total length (≥0 bp)	26,468,106
GC (%)	49.06
N50	1,540,514
N90	394,453
L50	7
L90	20
# N’s per 100 kbp	0.00
# of rRNA	42
# of tRNA	177
Complete BUSCOs (C) *	4653 (95.7%)
Complete and single-copy BUSCOs (S) *	4615 (94.9%)
Complete and duplicated BUSCOs (D) *	38 (0.8%)
Fragmented BUSCOs (F) *	43 (0.9%)
Missing BUSCOs (M) *	166 (3.4%)
Total lineage BUSCO *	4862

* Results obtained using “*Onygenales*_odb10” dataset; ** in units.

**Table 3:** PFAM domains associated with keratin degradation identified in different members of the Eurotiomycetes.

PFAM Domain			Endoproteases Catalyzing Keratin Hydrolysis				Exoproteases Involved in Keratin Hydrolysis				Oligopeptidases Involved in Keratin Hydrolysis	Other Enzymes Involved in Keratin Hydrolysis (Membrane Proteases)	
			Subtilase Family (PF00082)	Lon Protease (S16) C-terminal Proteolytic Domain (PF05362)	Insulinase (Peptidase Family M16) (PF00675)	Fungalysin Metallopeptidase (M36) (PF02128)	Prolyl Oligopeptidase Family (PF00326)	Serine Carboxypeptidase (PF00450)	Zinc Carboxypeptidase (PF00246)	Peptidase Family M28 (PF04389)	Peptidase Family M3 (PF01432)	Peptidase Family S41 (PF03572)	Metallopeptidase Family M24 (PF00557)
***Onygenales***	*Onygenaceae*	*Coccidioides immitis* RS	18	2	6	2	5	10	3	9	2	1	9
		*C. posadasii* C735	17	2	6	2	5	11	3	9	2	1	9
		*Uncinocarpus reesii* UAMH 1704	21	2	6	2	4	7	4	10	3	2	9
		*Aphanoascus verrucosus* IHEM 4434	21	2	6	2	5	8	4	12	2	1	9
		*Chrysosporium keratinophilum* CBS 104.62	22	2	6	2	5	8	4	11	2	1	9
	*Malbrancheaceae*	*Malbranchea zuffiana* CBS 219.58	19	2	7	1	6	7	4	8	2	1	11
	*Arthrodermataceae*	*Trichophyton rubrum* CBS 118892	21	4	9	9	3	15	4	15	2	2	13
		*T. benhamiae* CBS 112371	17	2	7	5	4	11	5	11	2	2	8
		*Nannizzia gypsea* CBS 118893	15	2	7	5	5	12	4	11	2	3	9
		*Microsporum canis* CBS 113480	19	2	6	5	6	11	4	11	2	2	9
	*Ajellomycetaceae*	*Paracoccidioides brasiliensis* PB01	7	2	7	0	5	5	1	6	2	1	7
		*Histoplasma capsulatum* G186AR	8	2	7	0	5	7	1	7	2	1	9
***Eurotiales***	*Aspergillaceae*	*Aspergillus fumigatus* Af293	6	2	5	1	5	12	1	7	2	1	11
		*A. flavus* NRRL3357	6	2	6	2	7	12	2	14	4	5	12
		*A. nidulans* FGSC A4	5	2	6	0	8	5	2	7	0	0	11
***Arachnomycetales***	*Arachnomycetaceae*	*Arachnomyces peruvianus* gpAraPeru1.1	5	2	6	0	6	9	2	7	2	2	10

**Table 4:** PFAM domains associated with plant cell wall degradation identified in different members of the Eurotiomycetes.

PFAM domain			Cellulases											Xylanases			Pectinases		
			Cellulase (Glycosyl Hydrolase Family 5) (PF00150)	Glycosyl Hydrolase Family 3 N Terminal Domain (PF00933)	Glycosyl Hydrolase Family 3 C-terminal Domain (PF01915)	Glycosyl Hydrolases Family 6 (PF01341)	Glycosyl Hydrolase Family 45 (PF02015)	Glycosyl Hydrolase Family 7 (PF00840)	Glycosyl Hydrolases Family 8 (PF01270)	Glycosyl Hydrolase Family 9 (PF00759)	Glycosyl Hydrolase Family 10 (PF00331)	Glycoside Hydrolase Family 44 (PF12891)	Glycosyl Hydrolase Family 48 (PF02011)	Glycosyl Hydrolases Family 11 (PF00457)	Glycosyl Hydrolase Family 12 (PF01670)	Glycosyl Hydrolase Family 30 Beta Sandwich Domain (PF17189)	Pectate Lyase Superfamily Protein (PF12708)	Pectate Lyase (PF04431)	Pectinesterase (PF01095)
***Onygenales***	*Onygenaceae*	*Coccidioides immitis* RS	1	5	3	0	0	0	0	0	0	0	0	0	0	0	2	0	0
		*C. posadasii* C735	1	5	3	0	0	0	0	0	0	0	0	0	0	0	2	0	0
		*Uncinocarpus reesii* UAMH 1704	1	5	3	0	0	0	0	0	0	0	0	0	0	0	4	0	0
		*Aphanoascus verrucosus* IHEM 4434	2	6	3	0	0	0	0	0	0	0	0	0	0	0	3	0	0
		*Chrysosporium keratinophilum* CBS 104.62	3	6	3	0	0	0	0	0	0	0	0	0	0	0	3	0	0
	*Malbrancheaceae*	*Malbranchea zuffiana* CBS 219.58	2	7	3	0	0	0	0	0	0	0	0	0	0	0	3	0	0
	*Arthrodermataceae*	*Trichophyton rubrum* CBS 118892	2	5	4	0	0	0	0	0	0	0	0	0	0	0	2	0	0
		*T. benhamiae* CBS 112371	3	6	3	0	0	0	0	0	0	0	0	0	0	0	2	0	0
		*Nannizzia gypsea* CBS 118893	2	6	3	0	0	0	0	0	0	0	0	0	0	0	2	0	0
		*Microsporum canis* CBS 113480	3	6	3	0	0	0	0	0	0	0	0	0	0	0	2	0	0
	*Ajellomycetaceae*	*Paracoccidioides brasiliensis* PB01	3	4	3	0	0	0	0	0	0	0	0	0	0	0	8	0	0
		*Histoplasma capsulatum* G186AR	3	4	3	0	0	0	0	0	0	0	0	0	0	0	6	0	0
***Eurotiales***	*Aspergillaceae*	*Aspergillus fumigatus* Af293	13	17	15	1	0	0	0	0	0	0	0	3	0	0	13	8	6
		*A. flavus* NRRL3357	14	24	22	1	0	0	0	0	0	0	0	4	0	0	6	13	6
		*A. nidulans* FGSC A4	13	21	19	2	0	0	0	0	0	0	0	2	0	0	14	12	3
***Arachnomycetales***	*Arachnomycetaceae*	*Arachnomyces peruvianus* gpAraPeru1.1	18	18	15	3	4	0	0	0	0	0	0	2	0	0	4	8	2

**Table 5:** PFAM domains associated with production of secondary metabolites identified in different members of the Eurotiomycetes.

PFAM domain			PKS				NRPS		NRPS/PKS	Terpene		
			Acyl Transferase Domain (PF00698)	Beta-Ketoacyl Synthase, C-terminal Domain (PF02801)	Beta-Ketoacyl Synthase, N-terminal Domain (PF00109)	Chalcone and Stilbene Synthases, N-terminal Domain (PF00195)	Condensation Domain (PF00668)	AMP-Binding Enzyme (PF00501)	Phosphopantetheine Attachment Site (PF00550)	Terpene Synthase Family 2, C-terminal Metal Binding (PF19086)	Terpene Synthase, N-terminal Domain (PF01397)	Terpene Synthase Family, Metal Binding Domain (PF03936)
***Onygenales***	*Onygenaceae*	*Coccidioides immitis* RS	13	13	13	1	23	48	34	1	0	0
		*C. posadasii* C735	11	12	12	1	23	49	33	1	0	0
		*Uncinocarpus reesii* UAMH 1704	9	9	8	1	29	57	31	0	0	0
		*Aphanoascus verrucosus* IHEM 4434	7	8	8	1	25	49	26	1	0	0
		*Chrysosporium keratinophilum* CBS 104.62	10	11	11	1	29	51	30	1	0	0
	*Malbrancheaceae*	*Malbranchea zuffiana* CBS 219.58	14	14	14	0	37	55	47	6	0	0
	*Arthrodermataceae*	*Trichophyton rubrum* CBS 118892	13	16	17	1	48	78	51	3	0	0
		*T. benhamiae* CBS 112371	15	15	16	1	55	72	60	3	0	0
		*Nannizzia gypsea* CBS 118893	21	22	23	1	47	69	51	3	0	0
		*Microsporum canis* CBS 113480	25	25	28	1	59	80	68	5	0	0
	*Ajellomycetaceae*	*Paracoccidioides brasiliensis* PB01	5	5	5	0	13	43	13	1	0	0
		*Histoplasma capsulatum* G186AR	5	5	6	0	20	49	22	0	0	0
***Eurotiales***	*Aspergillaceae*	*Aspergillus fumigatus* Af293	16	17	18	0	54	74	59	1	0	0
		*A. flavus* NRRL3357	39	39	44	3	72	121	102	11	0	0
		*A. nidulans* FGSC A4	33	35	36	0	52	89	69	4	0	0
***Arachnomycetales***	*Arachnomycetaceae*	*Arachnomyces peruvianus* gpAraPeru1.1	29	30	31	0	65	87	77	7	0	0

## Data Availability

This Whole Genome Shotgun project has been deposited in DDBJ/ENA/GenBank, under the accession JARUPO000000000; BioSample SAMN34004600.

## Supplementary Materials

The following supporting information can be downloaded at: https://www.mdpi.com/article/10.3390/jof10090600/s1, Table S1: Biosynthetic gene clusters found in the genome of *Malbranchea zuffiana* CBS 219.58 previously reported in other fungal taxa. Figure S1. Identified biosynthesis gene clusters (BGCs) in *Malbranchea zuffiana* CBS 219.58. NRPS—non-ribosomal peptide synthetase clusters; T1PKS—type 1 polyketide synthase clusters; fungal-RiPP-like—fungal post-translationally modified peptide product-like; NRPS-like—non-ribosomal peptide synthetase-like cluster.
