# Supplementary material for: Genomic Sequencing and Functional Analysis of the Ex-Type Strain of Malbranchea zuffiana
Source: J Fungi (Basel). 2024 Aug 24;10(9):600. doi: 10.3390/jof10090600 (PMC11433161; doi:10.3390/jof10090600)
Supplement: Supplementary file 1 [file jof-10-00600-s001.zip › jof-3113911-supplementary.pdf]

Supplementary Material

Table S1: Biosynthetic gene clusters found in the genome of *Malbranchea zuffiana* CBS 219.58 previously reported in other fungi

Compound	Species	Biosynthetic class	Similarity (%)
Equisetin	*Fusarium heterosporum*	NRP + Polyketide	18
Choline	*Aspergillus nidulans* FGSC A4	NRP	100
Trypacidin	*Aspergillus fumigatus* Af293	Polyketide	35
Enniatin	*Fusarium equiseti*	NRP	100
Griseofulvin/epidechlorogriseofulvin/norlichexanthone/dehydrogriseofulvin/4-desmethylgriseofulvin/griseoxanthone B	*Penicillium aethiopicum*	Polyketide	9
Chaetoglobosin P/chaetoglobosin K/chaetoglobosin A	*Discosia rubi*	Alkaloid	25
Trichobrasilenol/xylarenic acid B/brasilane A/brasilane F/brasilane E/brasilane D	*Annulohypoxylon truncatum*	Terpene	40
Squalestatin	*Aspergillus* sp. Z5	Terpene	40
Epichloenin A	*Epichloe festucae*	NRP	100
HEx-pks23	*Endocarpon pusillum* Z07020	Polyketide	33
Clavaric acid	*Hypholoma sublateritium*	Terpene	100
Solanapyrone A	*Ascochyta rabiei*	Polyketide	40
Communesin A/communesin B/communesin C/communesin D/communesin E/communesin G/communesin H	*Penicillium expansum*	Polyketide	25

NRP; Nonribosomal peptide

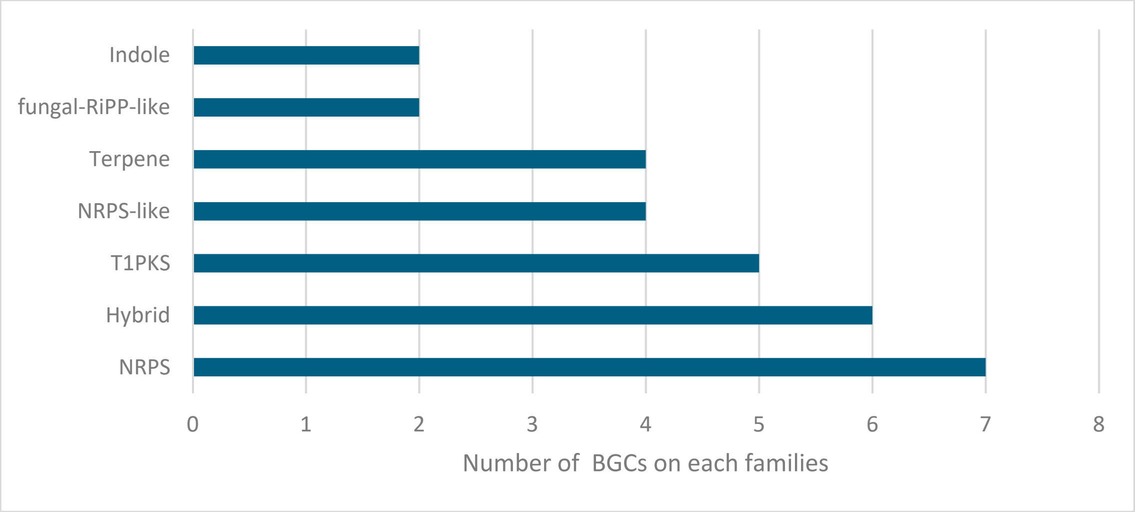

Imagen S1. Identified biosynthesis gene clusters (BGCs) in *Malbranchea zuffiana* CBS 219.58. (NRPS) non-ribosomal peptide synthetase clusters, (T1PKS) type 1 polyketide synthase clusters, (fungal-RiPP-like) Fungal post-translationally modified peptide product-like and (NRPS-like) non-ribosomal peptide synthetase-like cluster.
